# Adverse Events and Toxicity of Systemic Treatments for Uveal Melanoma: A Systematic Review

**DOI:** 10.3390/cancers18050781

**Published:** 2026-02-28

**Authors:** Katia Lanzafame, Giusi Blanco, Sabrina Paratore, Maria Grazia Maratta, Angela Russo, Salvatore Asero, Maria Gaetana Ursino, Paola Marino, Roberto Bordonaro

**Affiliations:** 1 Medical Oncology Unit, Department of Oncology, ARNAS Garibaldi Hospital, 95122 Catania, Italy; gblanco@arnasgaribaldi.it (G.B.); mursino@arnasgaribaldi.it (M.G.U.); rbordonaro@arnasgaribaldi.it (R.B.); 2 Human Pathology Unit, Department of Oncology, ARNAS Garibaldi Hospital, 95122 Catania, Italy; sparatore@arnasgaribaldi.it (S.P.); angelarct@tiscali.it (A.R.); 3 Department of Medical Oncology, Policlinico Gemelli, 00168 Rome, Italy; mariagrazia.maratta@guest.policlinicogemelli.it; 4 Surgical Oncology Unit, Department of Oncology, ARNAS Garibaldi Hospital, 95122 Catania, Italy; sasero@arnasgaribaldi.it; 5 Department of Human Pathology “G. Barresi”, School of Specialization in Medical Oncology, University of Messina, 98124 Messina, Italy; paolamarino43@gmail.com

**Keywords:** uveal melanoma, adverse events, toxicity, safety, chemotherapy, MEK inhibitor, bispecific drug, immunotherapy, antibody–drug conjugate

## Abstract

**Background/Objectives**: The primary objective of this systematic review is to synthesize the available evidence regarding the safety of the various treatment options for advanced uveal melanoma. A thorough understanding of a drug’s safety profile enables early identification and management of adverse reactions, thereby preventing clinical deterioration and minimizing the need for dose reduction, treatment delays, or therapy discontinuation. **Methods**: In accordance with the PRISMA (Preferred Reporting Items for Systematic Reviews and Meta-Analyses) and AMSTAR (Assessing the Methodological Quality of Systematic Reviews) guidelines, this review included all clinical studies that examined the most common adverse events associated with all available systemic treatments for metastatic uveal melanoma. Following the study selection process, nine studies were considered eligible and were included in the review. **Results**: Treatment with the bispecific antibody was associated with a favorable toxicity profile. The most severe adverse event observed was limited to cutaneous toxicity. Analysis of treatment-related adverse events (TRAEs) of grade ≥3 showed that patient cohorts receiving trametinib, selumetinib, and darovasertib experienced the lowest incidence of severe events (with the exception of creatine phosphokinase elevation observed with selumetinib), suggesting a comparatively more favorable safety profile for these agents. At present, the most robust efficacy data in the metastatic uveal melanoma setting are available for tebentafusp and darovasertib. **Conclusions**: This study provides the most comprehensive analysis of TRAEs in randomized trials of UM, delineating the toxicity and safety profiles of current therapies to guide personalized treatment decisions.

## Simple Summary

Uveal melanoma is a highly aggressive malignancy associated with a poor prognosis. Systemic therapeutic approaches for uveal melanoma encompass chemotherapy, immunotherapy, targeted therapies, bispecific antibodies, antibody–drug conjugates, and combination treatment regimens. The primary objective of this systematic review is to synthesize the available evidence regarding the safety profiles of these therapeutic strategies in advanced uveal melanoma, thereby providing clinicians with a comprehensive and authoritative overview of the current evidence base.

## 1. Introduction

Uveal melanoma (UM) is the most common primary intraocular malignancy in adults, arising from the choroid (85–90%), iris (3–5%), or ciliary body (5–8%) [1]. Although it represents only approximately 5% of all human melanomas, UM is the most frequent form of melanoma at non-cutaneous sites [2,3]. The incidence of UM in Europe has remained largely stable over time, exhibiting a decreasing North–South gradient—from 0.8 to 0.2 per 100,000—which is related to the higher prevalence of fair-skinned individuals with lightly pigmented irises in northern countries [4].

The principal risk factors for UM include fair skin, light-colored eyes, congenital ocular melanocytosis, ocular melanocytoma, and BAP1 tumor predisposition syndrome [3]. Most patients present with intraocular disease; however, approximately 3% have distant metastases at diagnosis, predominantly in the liver [5]. The risk of metastasis varies according to disease stage, with a 5-year risk ranging from 3–5% for stage I, 15–30% for stage II, and 44% or higher for stage III [6]. Notably, disease-specific survival (DSS) across all UM cases is estimated at 78.8% at 5 years, whereas for patients with metastatic disease, DSS decreases to below 20% (59.3%), a figure that has not improved significantly over recent decades [7].

In UM, most tumors—around 90%—harbor mutations in the G-protein subunits GNAQ or GNA11, while tumors without these mutations often carry alterations in CYSLTR2 or PLCB4. Experimental studies suggest that GNAQ/11 mutations are an early event in tumor development, but additional changes, such as loss of the tumor suppressor TP53, are required for full oncogenic transformation. Later mutations in genes like EIF1AX, BAP1, SF3B1, and SRSF2 further shape the tumor’s behavior, with BAP1 mutations combined with chromosome 3 loss (monosomy 3) being strongly associated with poor prognosis. Large-scale genomic analyses, such as those by TCGA, identified four distinct tumor clusters with varying outcomes, where clusters with BAP1 mutations and monosomy 3 had the worst prognosis and distinct molecular profiles. These high-risk tumors often show strong immune infiltration, including macrophages and high HLA expression, which correlates with increased metastasis and lower long-term survival [8].

The primary treatment for localized forms of UM is radiotherapy [9], including plaque brachytherapy or particle beam radiotherapy. Alternative options for patients who are not candidates for these therapies include laser treatment or enucleation. In cases of insufficient response to brachytherapy or particle beam radiotherapy, interventions such as surgical resection, laser ablation, transpupillary thermotherapy (TTT), or cryotherapy may be considered [10].

Common strategies for the management of distant metastases include surgery, radiotherapy (RT), ablative techniques, vaccines, localized chemotherapy or immunotherapy, and systemic therapies, such as chemotherapies, immunotherapies, targeted agents, bispecific antibodies, antibody–drug conjugates (ADCs), and combination regimens [11,12]. These treatment modalities have demonstrated limited efficacy and are associated with non-negligible adverse effects. The primary objective of our systematic review is to summarize the available evidence regarding the safety of these therapeutic approaches in advanced UM, providing clinicians with a comprehensive overview of the topic.

## 2. Materials and Methods

An iterative search process, informed by the results of the initial screening and refined through successive searches and discussions, was used to develop the search strategy. The systematic review was conducted in accordance with the Preferred Reporting Items for Systematic Reviews and Meta-Analyses (PRISMA) 2020 statement (Supplementary Materials) and the AMSTAR (Assessing the Methodological Quality of Systematic Reviews) guidelines.

The studies were defined according to the PICO strategy. The population of interest (P) included patients with advanced uveal melanoma. The intervention (I) consisted of pharmacological treatments, while the comparison (C) involved different therapeutic approaches, including chemotherapy, immunotherapy, ADCs, and bispecific antibodies. The outcomes (O) focused on toxicity and safety. Based on this framework, the research question was formulated as follows: What is the toxicity profile of drugs commonly used in advanced uveal melanoma, and which treatments are best tolerated?

The validated search strategy was applied to MEDLINE, the Cochrane Database, and Embase, with searches conducted within article titles, abstracts, and keywords.

The systematic review included all clinical studies analyzing the most common adverse events of available systemic treatments for metastatic UM. We systematically searched MEDLINE, Cochrane Database and Embase using the following keywords: “Uveal melanoma” AND “chemotherapies” OR “immunotherapies” OR “targeted therapies” OR “ADCs” OR “bispecific antibodies” AND “adverse events”. Studies were considered eligible in our systematic reviews if they met the following criteria: (1) clinical studies evaluating adverse events of systemic treatments for UM; (2) retrospective and prospective studies; (3) studies published in English only; (4) only articles with full-text were included. The exclusion criteria were: (1) inappropriate study design (conference abstracts, letters to editor and ongoing randomized trial), (2) preclinical studies, (3) duplicate publication or provision of insufficient data, (4) studies evaluating the adverse reactions of treatments in settings other than uveal melanoma.

The screening process started with the removal of duplicate records. The remaining studies were then independently screened in pairs through title and abstract review, in accordance with the predefined exclusion criteria. Articles considered potentially eligible were subjected to full-text assessment, together with additional studies identified through manual searching. Finally, the authors discussed the selected articles, reached consensus, and approved the studies included in the development of this review.

The methodological quality and risk of bias of the studies were assessed using the Newcastle–Ottawa Scale [13], which evaluates three domains: selection, comparability, and outcome (or exposure in case–control studies), the nine studies have a score of 5–7.

This paper was registered at PROSPERO, under number CRD420261296356.

## 3. Results

**Figure 1:**
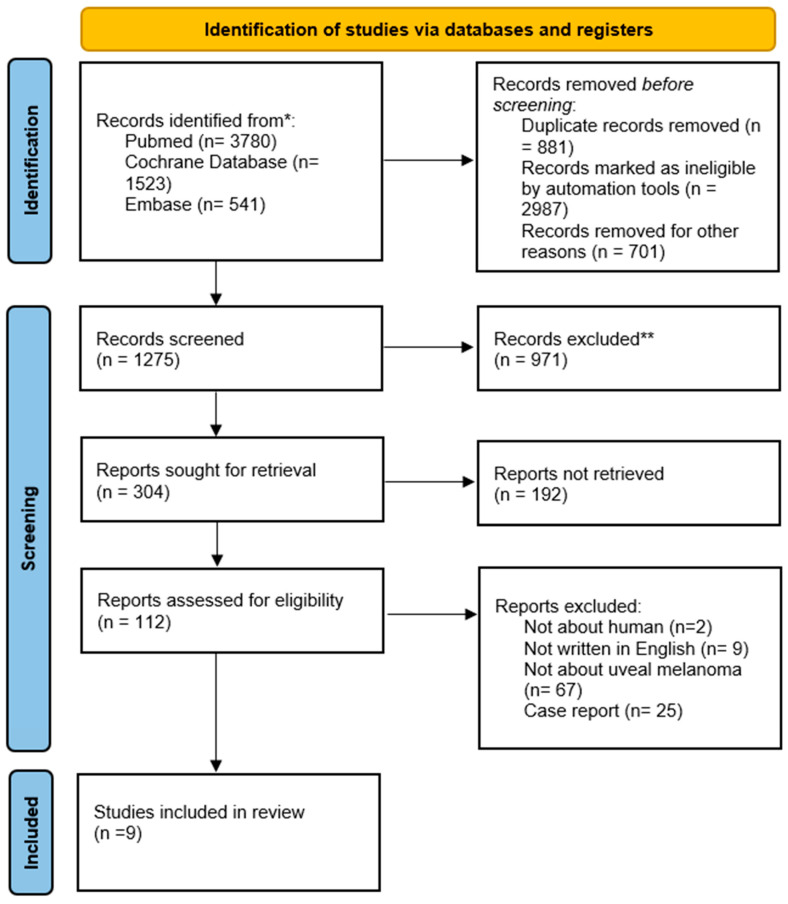
PRISMA flow chart (2020). ** not related to the treatment of uveal melanoma.

The initial search identified a total of 5844 studies. After removal of duplicate records, 4963 articles remained for the preliminary screening of titles and abstracts. Following this assessment, 112 studies were considered potentially eligible. Of these, 103 studies were excluded for the reasons outlined in the flowchart, resulting in a total of 9 studies included in the review (Figure 1).

The nine included studies collectively involved 896 participants. Of these, two were phase 3 trials, six were phase 2 trials and one multicenter expanded access program, published between 2010 and 2023. Comprehensive details regarding study settings, treatment, and efficacy outcomes are summarized in Table 1.

### 3.1. Bispecific Fusion Protein

Tebentafusp is a bispecific fusion protein designed to target the melanoma-associated antigen glycoprotein 100 (gp100) via a high-affinity T-cell receptor (TCR) binding domain. Concurrently, its anti-CD3 T-cell engaging domain recruits and activates T cells to mediate the cytotoxic elimination of gp100-expressing tumor cells. In a phase III randomized trial, treatment with tebentafusp resulted in improved overall survival compared with control therapy among previously untreated HLA-A*02:01-positive patients with metastatic uveal melanoma (UM). A total of 378 patients were randomized in a 2:1 ratio to receive either tebentafusp or a comparator therapy, which included single-agent pembrolizumab, ipilimumab, or dacarbazine, as determined by the investigator’s choice. After 36 months of follow-up, median overall survival was 21.6 months in the tebentafusp group versus 16.9 months in the control group (hazard ratio [HR] for death, 0.68; 95% confidence interval [CI], 0.54–0.87) [14,23].

The most frequently observed treatment-related adverse events (TRAEs) of any grade in the tebentafusp group were associated with cytokine release, including skin rash—attributable to gp100 expression in normal melanocytes—pyrexia, chills, and hypotension due to T-cell activation. Grade 3–4 adverse events included skin rash and elevated transaminases, particularly aspartate aminotransferase (AST). These TRAEs were most commonly observed during the first four weeks, coinciding with the dose-escalation phase, and generally within the first three months of treatment. Cytokine release syndrome was reported in up to 80–90% of patients receiving tebentafusp. Symptomatic management with antipyretics, glucocorticoids, and intravenous fluids is strongly recommended to mitigate these effects. Normally, infusions should not be permanently discontinued, but they can be delayed or reduced [24].

### 3.2. Immunotherapies

Nivolumab combined with ipilimumab represented the first immunotherapy regimen to demonstrate efficacy in previously treated patients with metastatic UM in a phase II study conducted by Pelster et al. [15] in 2021. Approximately 80% of participants experienced TRAEs, the most common being diarrhea, elevated liver enzymes, hypothyroidism, and pruritus. Grade 3–4 TRAEs were observed in 40% of patients, while no treatment-related deaths were reported.

The efficacy and safety of ipilimumab as a single agent were also evaluated in a separate phase II trial conducted by the Dermatologic Cooperative Oncology Group (DeCOG). This trial enrolled both pretreated and treatment-naïve patients with metastatic UM and demonstrated limited clinical activity. Approximately 66% of treated patients experienced TRAEs of any grade, with 36% classified as grade 3–4. The most frequent grade 3–4 immune-related adverse events (irAEs) were colitis and diarrhea. Notably, one potential treatment-related death due to pancytopenia, complicated by subsequent cerebral hemorrhage and respiratory insufficiency, was reported [25].

Danieli et al. [16] conducted an additional study evaluating ipilimumab in previously treated patients with metastatic UM. The therapy failed to elicit any objective tumor responses. Furthermore, 23% of participants experienced grade ≥3 adverse events, specifically thrombocytopenia, diarrhea, and elevated liver enzymes. Overall, 18 cases of immune-related adverse events (irAEs) of any grade were recorded, affecting the skin, gastrointestinal tract, liver, endocrine system, hematologic parameters, and other organ systems. No grade 4 irAEs were reported.

The management of immune-related adverse events depends on their severity: in mild cases (grade 1), therapy can be continued with simple monitoring; in moderate cases (grade 2), therapy is temporarily suspended and oral corticosteroids are administered; in severe cases (grade 3 or 4), treatment is discontinued and high-dose corticosteroids are used, possibly with additional immunosuppressants such as infliximab for refractory colitis or mycophenolate for hepatitis. Endocrinopathies, such as thyroiditis or hypophysitis, often require only replacement therapy and do not necessarily require discontinuation of immunotherapy [26].

### 3.3. Chemotherapies

Systemic chemotherapy is generally ineffective in metastatic UM. Consequently, chemotherapy is not typically considered a first-line treatment for UM; however, it may be employed in cases where the disease has metastasized and alternative therapeutic approaches are unavailable or have failed. Various chemotherapeutic agents have been utilized in the management of metastatic UM, largely based on experience derived from cutaneous melanoma, such as dacarbazine [27]. Evidence regarding the use of chemotherapy in this context is limited, and no agent has been shown to improve survival in patients with metastatic UM, as prospective, randomized phase III trials have not been conducted in this setting.

Only a single phase II clinical trial has evaluated the efficacy and safety of DHA-paclitaxel in patients with UM. In this study, 32% of treated patients achieved stable disease, with a median overall survival (OS) of 9.8 months. The drug exhibited a favorable toxicity profile, with Grade 3–4 neutropenia observed in 23% of patients, musculoskeletal pain in 10%, and rash in 5%. The most common adverse events of any grade included constipation (55%), fatigue (64%), headache (64%), myalgia (64%), nausea (55%), pain (73%), and neuropathy (motor or sensory) (23%) [17].

Although systemic chemotherapy yields minimal response rates, transarterial chemoembolization appears to have some efficacy in the management of hepatic metastases. S. Leyvraz et al. [18] demonstrated that, in patients with liver metastases from UM, hepatic intra-arterial administration of fotemustine did not result in improved overall survival compared with systemic therapy. The most frequent grade ≥3 toxicities were thrombocytopenia and neutropenia.

### 3.4. Targeted Therapies

Although BRAF mutations are infrequent in UM, most of these tumors harbor mutations in GNAQ or GNA11 [28]. These alterations result in constitutive activation of the RAS/RAF/MEK/ERK signaling pathway, which is critically involved in regulating cell proliferation and survival [29]. The persistent activation of this pathway represents a central driver of UM development and progression, thereby constituting a potential target for therapeutic intervention.

Selumetinib is a selective, non-adenosine triphosphate (ATP)-competitive inhibitor of MEK1 and MEK2, key kinases within the RAS/RAF/MEK/ERK pathway. In a randomized clinical trial conducted by Carvajal et al. [19], selumetinib demonstrated a modest improvement in PFS and objective response rate relative to conventional chemotherapy in patients with metastatic UM. Median overall survival (OS) was 9.1 months in the chemotherapy group compared to 11.8 months in the selumetinib-treated cohort. Adverse events of any grade were observed in 97% of patients receiving selumetinib. The most commonly observed adverse effects included acneiform rash (Grade 1–2: 76%; Grade 3: 2%), CPK elevation (Grade 1–2: 37%; Grade 3: 16%), fatigue (Grade 1–2: 61%; Grade 3: 0%), and transaminase elevation (Grade 1–2: 41%; Grade 3: 10%). Grade 3–4 events were reported in 47% of patients (*n* = 23). Visual disturbances, including blurred vision, were also observed (Grade 1–2: 8%). The control group received temozolomide as chemotherapy. In this group, the most frequent adverse events were constipation (Grade 1–2: 30%), fatigue (Grade 1–2: 44%), anemia (Grade 1–2: 16%), neutropenia (Grade 1–2: 8%; Grade 3: 2%), thrombocytopenia (Grade 1–2: 16%), nausea (Grade 1–2: 40%), and vomiting (Grade 1–2: 24%).

In 2016, the combination of a protein kinase B (PKB/AKT) inhibitor with trametinib was evaluated in patients with uveal melanoma (UM). This combination did not demonstrate improvement in PFS in UM patients; however, several TRAEs were reported. Trametinib-associated adverse events included rash (100% of patients, Grade 1–2), diarrhea (72%, Grade 1–2), elevated transaminases (mild in 55% of patients; Grade 3 in 6%), and mucositis (50%, Grade 1–2). Seven patients required dose reductions due to these side effects [20].

The high prevalence of GNAQ/GNA11 mutations prompted the development of a novel class of agents, including the oral protein kinase C (PKC) inhibitor darovasertib. A Phase I trial enrolled 68 patients with metastatic UM. Darovasertib demonstrated an objective response rate (ORR) of 9.1% (1 complete response and 5 partial responses) and a disease control rate (DCR) of 75% (45 patients achieved stable disease as the best response) [30]. The median duration of response was 10.1 months. Regarding tolerability, 42.6% of patients experienced Grade 3–4 TRAEs. Among pretreated patients with metastatic melanoma, 30% achieved a partial response, increasing to 45% in treatment-naive patients; median PFS was 7 months in both groups. Clinical efficacy was observed in both HLA-A2-positive and -negative patients [21].

Based on these favorable efficacy results, a Phase II/III clinical trial is currently underway to evaluate the combination of darovasertib and crizotinib in first-line HLA-A*02:01-negative patients.

### 3.5. Antibody–Drug Conjugate (ADC)

Glembatumumab vedotin is an antibody–drug conjugate targeting the gpNMB protein and linked to the potent cytotoxic microtubule inhibitor, MMAE. MMAE (payload) blocks cell division by inhibiting microtubules, but if released outside tumor cells or in normal tissues, it can cause toxicity, including neuropathy and myelosuppression. Meanwhile, gpNMB is the antibody’s target protein: its high expression in tumors enhances the ADC’s efficacy, while its presence in normal tissues contributes to side effects. In summary, the efficacy and toxicity of an ADC depend on the interaction between the potent MMAE payload and the distribution of the gpNMB target. A Phase II study investigated the efficacy and safety of glembatumumab vedotin in patients with metastatic UM. A total of 35 chemotherapy-naïve patients were enrolled. GPNMB expression was assessed immunohistochemically both prior to and following therapy. The study reported an mPFS of 3.1 months and a mOS of 11.9 months. Among the participants, 6% achieved a partial response (PR), while 51% demonstrated stable disease (SD) as their best response. Alopecia, maculopapular rash, pruritus, leukopenia, neutropenia, anemia, elevated ALT/AST, nausea, diarrhea, fatigue and peripheral neuropathy were the most frequently observed treatment-related adverse events (TRAEs). Grade 3/4 TRAEs were primarily neutropenia (48%) and were generally manageable, while other severe events occurred in ≤6% of patients. A single grade 5 event of encephalopathy was reported, attributed to hepatic disease progression rather than treatment. Overall, 27 serious adverse events (SAEs) were recorded, most commonly neutropenia and organ toxicities, with a grade 5 SAE incidence of 3% [22].

## 4. Discussion

Immunotherapies, chemotherapies, targeted therapies, bispecific fusion proteins, ADCs, and systemic therapy combinations have been evaluated in prospective trials for the treatment of metastatic uveal melanoma (UM). We examined the TRAEs and their incidences as reported in studies of drugs administered to patients with UM. Hematologic toxicities—namely anemia, neutropenia, and thrombocytopenia—were more frequent in patients receiving chemotherapy and ADCs [17,22]. Fotemustine, in particular, was associated with high rates of severe anemia (87.9%), neutropenia (62.9%), and thrombocytopenia (42.1%) [18].

Gastrointestinal and dermatologic adverse events, as well as hypothyroidism and elevated liver enzymes, were more common among patients treated with PD-L1 and/or CTLA-4 inhibitors. Combination therapy with nivolumab and ipilimumab resulted in a 17% incidence of grade 3–4 liver enzyme elevation [25]. Ipilimumab monotherapy was associated with a notable incidence of colitis, with 13% of cases being severe [16].

MEK inhibitors (trametinib and selumetinib) exhibited a similar toxicity profile [19,20], with skin rash being the most frequent adverse event and only a few cases of severe presentation. Fatigue was reported in 61% of patients treated with selumetinib, compared with 31% of those receiving trametinib.

Treatment with bispecific antibodies was associated with high rates of adverse events of any grade, including skin rash (83%), chills (49%), fever (76%), pruritus (76%), fatigue (42%), and hypotension (38%). Grade 3 skin rash occurred in 19% of cases.

Table 2 summarizes the TRAEs associated with all therapies in metastatic UM, with particular emphasis on the rates of severe adverse events (grade 3–4).

**Figure 2:**
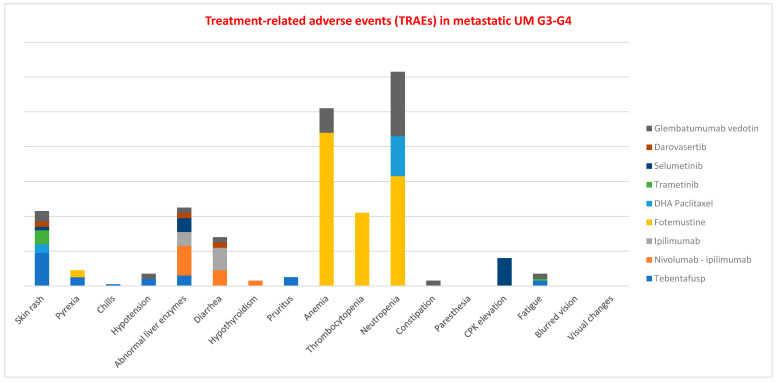
The figure shows the sum of the percentages of adverse events (TRAEs) of G3-G4 for the different drug classes. Neutropenia was one of the most common G3-G4 side effects along with anemia, abnormal liver enzymes and skin rash. High rates of neutropenia and anemia reported with chemotherapy and Glembatumumab vedotin. The combination of nivolumab and ipilimumab has reported high rates of abnormal liver enzymes G3-G4. Other drugs responsible for this alteration are: ipilimumab monotherapy, selumetinib, and tebentafusp. Skin rash is mainly caused by tebentafusp, followed by trametinib, Glembatumumab vedotin, DHA–paclitaxel, and selumetinib, darovasertib.

The cumulative percentages of grade 3–4 treatment-related adverse events (TRAEs) across different drug classes are illustrated in the analysis (Figure 2). Neutropenia was among the most frequent severe adverse events, along with anemia, elevated liver enzymes, and skin rash. Particularly high rates of neutropenia and anemia were observed with chemotherapy and Glembatumumab vedotin. The combination of nivolumab and ipilimumab was associated with a notable incidence of grade 3–4 liver enzyme abnormalities, while additional agents contributing to hepatic alterations included ipilimumab monotherapy, selumetinib, and tebentafusp. Skin rash was predominantly reported with tebentafusp, followed by trametinib, Glembatumumab vedotin, DHA-paclitaxel, selumetinib, and darovasertib.

**Figure 3:**
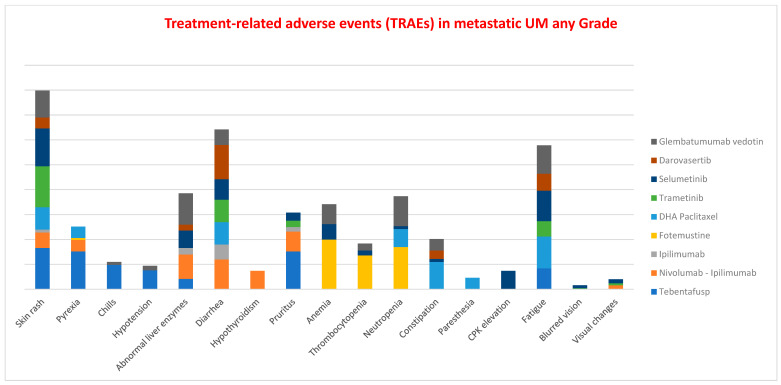
The figure shows the sum of the percentages of adverse events (TRAEs) of each grade for the different drug classes. The highest column is that of skin rash, followed by diarrhea, fatigue and abnormal liver enzymes. For tebentafusp, skin toxicity is comparable (any grade) to that of trametinib, and fatigue is reported in lower percentages than with chemotherapy and Glembatumumab vedotin. Diarrhea is reported with similar frequency between the patients treated with darovasertib and nivolumab and ipilimumab.

The distribution of TRAEs of each grade (Figure 3) across the various drug classes reveals that skin rash was the most common adverse event, followed by diarrhea, fatigue, and abnormal liver enzymes. For patients treated with tebentafusp, the incidence of skin toxicity (all grades) was comparable to that observed with trametinib, while fatigue occurred less frequently than with chemotherapy or Glembatumumab vedotin. Diarrhea was reported at similar rates among patients receiving darovasertib and those treated with the nivolumab–ipilimumab combination.

Our systematic review enabled an indirect comparison of immunotherapies, targeted therapies, bispecific antibodies, ADCs, and chemotherapy. Treatment with the bispecific antibody was associated with a favorable toxicity profile, with the most severe adverse event reported being limited to skin toxicity. Analysis of TRAEs of grade ≥3 indicated that populations receiving trametinib, selumetinib, or darovasertib experienced the lowest number of severe events (with the exception of CPK elevation observed with selumetinib), suggesting a more favorable safety profile for these therapies. The most robust efficacy data in the metastatic setting of uveal melanoma (UM) are currently available for tebentafusp and darovasertib. A detailed evaluation of toxicity profiles is essential for clinical practice and optimal patient management.

To our knowledge, this work represents the comprehensive analysis of TRAEs from randomized trials available for UM. It should be noted that the trials included in this analysis enrolled heterogeneous patient populations (treatment-naive and previously treated patients), and differences in follow-up duration across studies may have limited the detection of late-onset adverse events. Access to individual patient data would be necessary to overcome these limitations, providing more precise information regarding the adverse events under investigation. Moreover, the included studies were heterogeneous in terms of phase: two were phase 3 trials, six were phase 2 trials, and one was a multicenter expanded access program. This variability may also represent a potential source of bias.

Our aim was to define the toxicity and safety profile of treatments currently used for metastatic UM, thereby raising awareness of these issues and assisting clinicians in tailoring therapy to individual patient characteristics and comorbidities. Moreover, knowledge of a drug’s safety profile facilitates early identification and management of adverse reactions, preventing clinical deterioration, dose reductions, treatment delays, or even discontinuation.

## 5. Conclusions

High metastatic potential, limited responsiveness to conventional therapies, and the distinct molecular signature of uveal melanoma (UM) render it a significant clinical challenge. Immunotherapies, chemotherapies, targeted therapies, bispecific fusion proteins, antibody–drug conjugates (ADCs), and combinations of systemic therapies have been evaluated in prospective trials for the treatment of metastatic UM. This systematic review summarizes the current evidence regarding the safety of these therapeutic approaches in advanced UM, providing clinicians with a comprehensive overview of the topic.

## Figures and Tables

**Table 1:** Characteristics of the studies.

	N. Patients	Phase	Setting	Treatment	Evolution
**Jessica C. Hassel et al. (2023) [14]**	378 patients (tebentafusp, *n* = 252) (pembrolizumab or ipilimumab or dacarbazine, *n* = 126)	Randomized (2:1), open-label, phase 3	Previously untreated metastatic	Tebentafusp	- mOS was 21.6 months in the tebentafusp group and 16.9 months in the control group.
**PELSTER M.S. et al. (2021) [15]**	33 patients	Open-label, phase 2	Chemotherapy-naive and previously treated patients metastatic	Nivolumab and Ipilimumab	- mPFS was 5.5 months. - mOS was 19.1 months.
**Danielli R. et al. (2012) [16]**	13 patients	Multicenter expanded access program (EAP)	Previously untreated or treated metastatic (no prior treatment with ipilimumab)	Ipilimumab	- no objective responses. - mOS was 36 weeks.
**Homsi J. et al. (2010) [17]**	22 patients	Open-label, phase 2	Chemotherapy-naive and previously treated patients metastatic	DHA Paclitaxel	- Seven patients (32%) had stable disease with a median duration of 3 months. - The median overall survival was 9.8 months.
**Leyvraz S. et al. (2014) [18]**	171 patients hepatic intra-arterial (HIA) versus systemic (IV) fotemustine	Randomized (1:1), open-label, phase 3	Previously untreated or treated patients with liver metastases from UM.	Fotemustine	- HIA mOS 14.6 months vs. IV arm mOS 13.8 months.
**Carvajal R.D. et al. (2014) [19]**	101 patients (selumetinib, *n* = 50) (temozolomide or dacarbazine *n* = 51)	Randomized (1:1), open-label, phase 2	Chemotherapy-naive and previously treated patients metastatic (prior therapy with an MEK inhibitor, temozolomide, or dacarbazine was not permitted)	Selumetinib	- mPFS among patients randomized to chemotherapy was 7 weeks and among those randomized to selumetinib was 15.9 weeks. - mOS time was 9.1 months with chemotherapy and 11.8 months with selumetinib.
**Shoushtari A.N. (2016) [20]**	80 patients (trametinib, *n* = 40) (trametinib + Akt inhibitor, *n* = 40)	Randomized (1:1), open-label, phase 2	Previously untreated metastatic	Trametinib	- Not difference in mPFS between trametinib + Akt inhibitor and trametinib
**M. McKean et al. (2023) [21]**	63 patients (Darovasertib and Crizotinib)	Open-label, phase ½	First-line and previously treated patients metastatic	Darovasertib and Crizotinib	- mPFS was 7 months. - In pts with hepatic-only disease, an ORR of 35% and mPFS was 11 months.
**Merve Hasanov et al. (2020) [22]**	35 patients	Open-label, phase 2	metastatic uveal melanoma who had not previously been treated with chemotherapy	Glembatumumab vedotin	- mPFS was 3.1 months. - mOS was 11.9 months.

**Table 2:** Treatment-related adverse events (TRAEs) in metastatic UM.

	Tebentafusp		Nivolumab Ipilimumab		Ipilimumab		Fotemustine		DHA Paclitaxel		Trametinib		Selumetinib		Darovasertib		Glembatumumab Vedotin	
	Any Grade	G3-4	Any Grade	G3-4	Any Grade	G3-4	Any Grade	G3-4	Any Grade	G3-4	Any Grade	G3-4	Any Grade	G3-4	Any Grade	G3-G4	Any Grade	G3-4
**Skin rash**	83%	19%	31%	0%	6%	0%	-	-	45%	5%	82%	8%	76%	2%	22.1%	3.2%	54%	6%
**Pyrexia**	76%	5%	23%	0%	-	-	3.6%	3.6%	23%	-	-	-	-	-	-	-	-	-
**Chills**	49%	1%	-	-	-	-	-	-	-	-	-	-	-	-	-	-	6%	0%
**Hypotension**	38%	4%	-	-	-	-	-	-	-	-	-	-	-	-	-	-	9%	3%
**Abnormal liver enzymes**	21%	6%	49%	17%	13%	8%	-	-	-	-	-	-	35%	8%	11.6%	3.2%	63%	3%
**Diarrhea**	-	-	60%	9%	30%	13%	-	-	45%	-	45%	0%	41%	0%	69.5%	3.2%	31%	3%
**Hypothyroidism**	-	-	37%	3%	0%	0%	-	-	-	-	-	-	-	-	-	-	-	-
**Pruritus**	76%	5%	40%	0%	9%	0%	-	-	-	-	13%	0%	16%	0%	-	-	-	-
**Anemia**	-	-	-	-	-	-	100%	87.9%	-	-	-	-	31%	0%	-	-	40%	14%
**Thrombocytopenia**	-	-	-	-	-	-	68%	42.1%	-	-	-	-	10%	0%	-	-	14%	0%
**Neutropenia**	-	-	-	-	-	-	85.5%	62.9%	36%	23%	-	-	6%	0%	-	-	60%	37%
**Constipation**	-	-	-	-	-	-	-	-	55%	-	-	-	6%	0%	16.8%	0%	23%	3%
**Paresthesia**	-	-	-	-	-	-	-	-	23%	-	-	-	-	-	-	-	-	-
**CPK elevation**	-	-	-	-	-	-	-	-	-	-	-	-	37%	16%	-	-	-	-
**Fatigue**	42%	3%	-	-	-	-	-	-	64%	-	31%	1%	61%	0%	33.7%	0%	57%	3%
**Blurred vision**	-	-	-	-	-	-	-	-	-	-	2%	0%	6%	0%	-	-	-	-
**Visual changes**	-	-	8%	0%	-	-	-	-	-	-	4%	0%	8%	0%	-	-	-	-

## Data Availability

No new data were created or analyzed in this study.

## Supplementary Materials

The following supporting information can be downloaded at: https://www.mdpi.com/article/10.3390/cancers18050781/s1, PRISMA checklist.

## Abbreviations

The following abbreviations are used in this manuscript:

ADC	Antibody–Drug Conjugate
AKT	Ak strain transforming
AMSTAR	Assessing the methodological quality of systematic reviews
Anti CD3	anti Cluster of Differentiation 3
Anti PDL1	programmed death ligand 1
AST	aspartate aminotransferase
BAP1 tumor	BRCA1-Associated Protein 1
CI	confidence interval
CPK	creatine phosphokinase
CTLA4	cytotoxic T-lymphocyte associated protein 4
DeCOG	Dermatologic Cooperative Oncology Group
DHA -paclitaxel	acid docosahexaenoic acid paclitaxel
DSS	Disease-specific survival
ERK	extracellular signal-regulated kinase pathway
GNA11	guanine Nucleotide-binding Protein, Alpha-11
GNAQ	guanine nucleotide-binding protein G(q) subunit alpha
GPNMB	Glycoprotein non-metastatic melanoma protein B
HLA* 02:01	human leukocyte antigen * 02:01
HR	hazard ratio
irAEs	immune-related adverse events
MEK 1-2	mitogen-activated protein kinase 1-2
MEK inhibitor	mitogen-activated protein kinase inhibitor
MMAE	Monomethyl Auristatin E
OS	overall survival
PFS	Progression-free survival
PKB	protein kinase B
PKC inhibitor	Protein Kinase C inhibitor
PRISMA	Preferred Reporting Items for Systematic Reviews and Meta-Analyses
RAF	rapidly accelerated fibrosarcoma
RAS	reticular activating system
RT	radiotherapy
TCR	T-cell receptor
TRAEs	treatment-related adverse events
TTT	transpupillary thermotherapy
UM	uveal melanoma
